# Diagnoses of obstetric and postpartum thyroid disease: a Danish validation study

**DOI:** 10.1038/s41598-024-59636-w

**Published:** 2024-04-16

**Authors:** Anne Myrup Houmøller, Katrine Gerlif, Nanna Maria Uldall Torp, Stine Linding Andersen

**Affiliations:** 1 Department of Clinical Biochemistry, Aalborg University Hospital, 9000 Aalborg, Denmark; 2 Department of Clinical Medicine, Aalborg University, 9000 Aalborg, Denmark

**Keywords:** Thyroid diseases, Epidemiology

## Abstract

Different diagnoses of thyroid disease are available in the 10th International Classification of Diseases (ICD-10), but the validity of diagnoses related to obstetric and postpartum thyroid disease is unknown. This was a retrospective cohort study of all patients in the North Denmark Region with a diagnosis of postpartum thyroiditis (PPT) (ICD-10: O905) from 2016 to 2019 or obstetric thyroid disease in 2019 (ICD-10: O992B (hypothyroidism) or O992C (hyperthyroidism)) registered in the Danish National Hospital Register. Information from nationwide registers and medical records were used to assess the validity. Among patients with an O905-diagnosis (n = 40), abnormal thyroid function test results were seen in all cases. A total of eight patients (20.0%) were positive for thyrotropin receptor antibodies postpartum, however, in low titers, and PPT was verified in 39 of 40 cases (97.5%). Altogether 45 of 50 patients with an O992B-diagnosis (90.0%) correctly had hypothyroidism, whereas hyperthyroidism was found in 25 of 39 patients with an O992C-diagnosis (64.1%). This is the first study to validate ICD-10 diagnoses of obstetric and postpartum thyroid disease. A high validity was seen for PPT (O905) and obstetric hypothyroidism (O992B), whereas for obstetric hyperthyroidism (O992C), the diagnosis could not be verified in one third of the cases.

## Introduction

Thyroid disorders are mainly of autoimmune origin in women of reproductive age^1^. During pregnancy, a normal maternal thyroid function is essential to prevent adverse obstetric outcomes and impaired fetal neurodevelopment^1^. After delivery, rebound of the immune system may trigger the onset of autoimmune thyroid disorders such as Graves’ disease (GD), Hashimoto’s thyroiditis (HT), and postpartum thyroiditis (PPT)^1^. PPT is a painless destructive thyroiditis defined as the development of de novo transient thyroid dysfunction within the first postpartum year in previously euthyroid women^2^. Women with non-thyroid autoimmune diseases, family history of autoimmune diseases, or a prior history of PPT have an increased risk of developing PPT^3,4^. The disorder may present with a classic biphasic presentation with initial thyrotoxicosis due to release of stored thyroid hormones from an inflamed gland followed by a hypothyroid phase lasting until euthyroidism reoccurs^5^. However, the clinical presentation may also be transient hypothyroidism alone or isolated transient thyrotoxicosis^1^. Most women return to the euthyroid state within one year postpartum, however, some women do not restore normal thyroid function after the initial episode of hypothyroidism^3^. PPT often remains asymptomatic but medical treatment may be indicated in case of notable symptoms^6^. In the thyrotoxic phase, antithyroid drugs (ATD) are not recommended but symptoms can be managed with beta-adrenergic agonists^6^. Levothyroxine may be used in the hypothyroid phase and tapering should be attempted after 6–12 months^6^. It is of great importance to distinguish PPT from other autoimmune thyroid disorders in the postpartum period, especially GD, as treatment differs^6^. For this purpose, thyroid peroxidase antibodies (TPO-Ab) and thyroglobulin antibodies (Tg-Ab) are less relevant as they often are present in both disorders. On the contrary, the detection of thyrotropin receptor antibodies (TRAb) is essential as elevated titers are highly sensitive and specific for GD, while women with PPT usually are TRAb negative^6^.

In Denmark, health data are systematically registered in nationwide registers, and the Danish National Hospital Register (DNHR) holds information on all hospital diagnosis registered according to the 10th International Classification of Diseases (ICD-10)^7^. Regarding thyroid disease, different ICD-10 diagnoses exist, and a certain group of diagnoses relates to obstetrics and the postpartum period, specifically. However, the validity of this subgroup of diagnoses remains unclear. The aim of this study was to review obstetric and postpartum thyroid diagnoses in the DNHR among patients in the North Denmark Region and to evaluate the validity of the diagnoses.

## Methods

### Study population

The study was a Danish retrospective cohort study of patients with hospital diagnoses of thyroid disease registered in relation to obstetrics or the postpartum period. In Denmark, women with thyroid disease who become pregnant are referred to hospital for management of the thyroid disease in an endocrine specialist department as well as augmented monitoring of maternal and fetal health in the obstetric department. In the postpartum period, women with known thyroid disease continue to be monitored via the endocrine specialist department in hospital whereas newly diagnosed postpartum thyroid disease can be managed in general practice or referred for endocrine specialist assessment depending on the severity of the disease.

**Figure 1:**
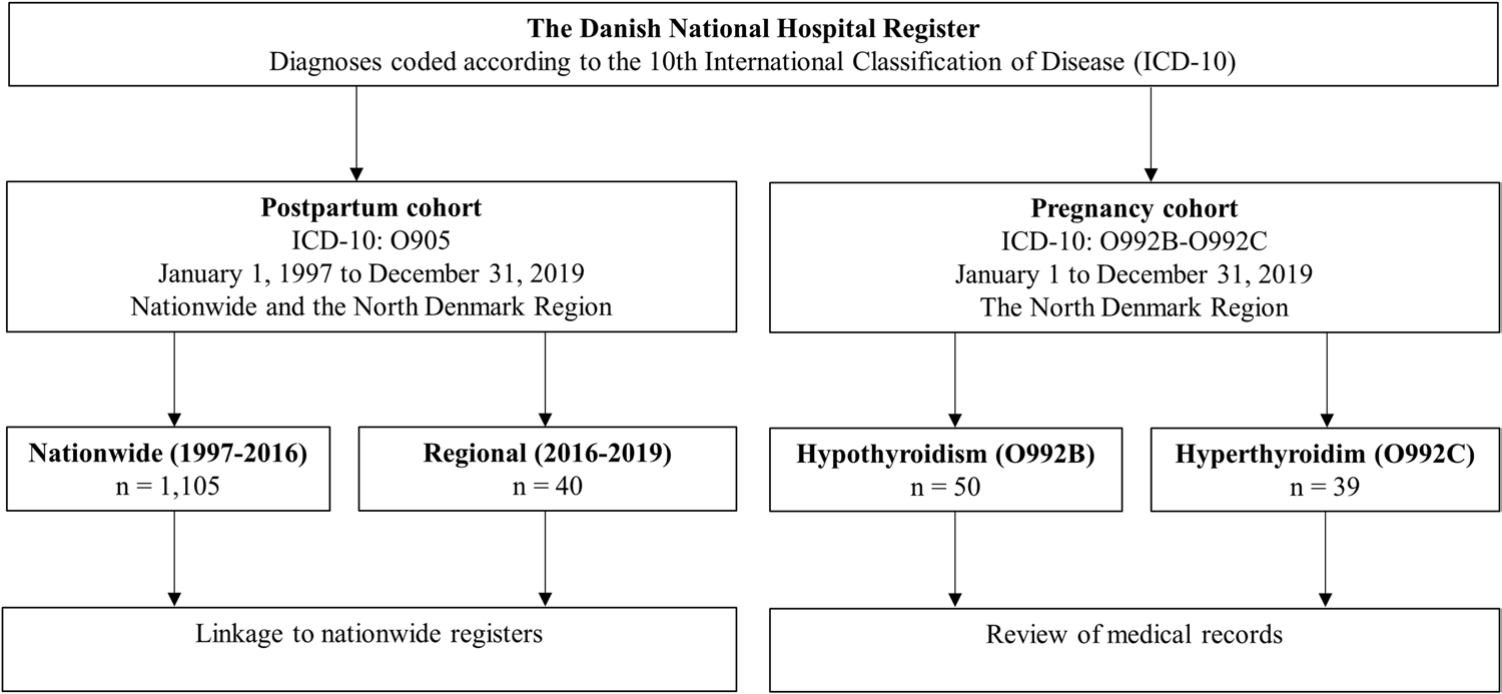
Flowchart of the study population including the postpartum and the pregnancy cohort.

The study population included women of reproductive age who were identified with specific diagnoses in the DNHR within the study period (Fig. 1). The DNHR holds all in- and outpatient diagnoses from Danish hospitals registered according to ICD-10^7^. In the DNHR, all women registered nationwide with a diagnosis of PPT (ICD-10: O905) in all regions of Denmark from 1997 to 2016 (n = 1,105) were identified (Fig. 1). Additionally, those who had the diagnosis registered at a hospital in the North Denmark Region from January 1, 2016, to December 31, 2019 (n = 40) were identified as a regional cohort. In total, these patients composed the postpartum cohort (Fig. 1). Furthermore, women with an ICD-10 diagnosis of O992B (Hypothyroidism complicating pregnancy, childbirth, or the puerperium) as well as O992C (Thyrotoxicosis complicating pregnancy, childbirth, or the puerperium) were identified in the North Denmark Region in 2019, and all women with a diagnosis of O992C this year were enrolled (n = 39) as well as a random sample of women with a diagnosis of O992B (n = 50) including a total of 89 women in the obstetric cohort (Fig. 1). The hospitals in the North Denmark Region approved the retrospective, observational study design with a waiver of patient informed consent. The Scientific Ethics Committee for the North Denmark Region deemed the study exempt from review. The study was registered according to the General Data Protection Regulation in the North Denmark Region (2021–077).

### Nationwide registers

In the postpartum cohort, information on maternal characteristics as well as thyroid and non-thyroid autoimmune diseases was gathered from DNHR, the Danish National Prescription Register (DNPR), and the Medical Birth Register (MBR) via Statistics Denmark. The DNPR holds information on all redeemed prescriptions of drugs from Danish pharmacies coded according to the anatomical therapeutic chemical (ATC) classification^8^, and the MBR holds information on all live- and still births in Denmark including information on a series of maternal characteristics (age, parity, smoking status, and pre-pregnancy body mass index (BMI))^9^. Information from DNHR and DNPR was collected to identify maternal known thyroid disease (ICD-10: E03 and E05, and ATC: H03A and H03B), and non-thyroid autoimmune diseases (rheumatoid arthritis: ICD-10 M05-06, inflammatory bowel disease: ICD-10 K50-51, and type 1 diabetes: ICD-10 E10 and ATC A10). In the obstetric cohort, information on maternal and disease characteristics was obtained via the retrospective review of patient medical records. This method of data collection allowed for the identification of information on treatment in hospital but did not include any treatment prescribed outside of hospital, e.g., by the general practitioner.

### Biochemical analyses

Among women in the postpartum cohort who had the diagnosis registered in the North Denmark Region from 2016 to 2019, biochemical results requested as part of routine clinical care were collected from the North Denmark Region Clinical Laboratory System II^10^. This laboratory system holds all routine biochemical results released from the regional Departments of Clinical Biochemistry and includes biochemical tests requested during patient hospital visits as well as visits in general practice. The biochemical results were registered for each patient during a two-year follow-up period from the date of childbirth or until a new pregnancy occurred, whichever came first. The biochemical measurements were performed using an automatic immunoassay for thyrotropin (TSH) (Cobas, Roche Diagnostics or Dimension Vista, Siemens Healthineers), total triiodothyronine (T3), and total thyroxine (T4) (Cobas, Roche Diagnostics or Centaur XPT, Siemens Healthineers), as well as TPO-Ab and Tg-Ab (Kryptor, Thermo Fisher Diagnostics). Finally, TRAb were measured using a radioimmunoassay (DYNOtest TRAK human, Brahms Diagnostica). Values below the lower measurement range were imputed as half the lowest measurable concentration (TSH: 0.005 mIU/L, TPO-Ab: 30 kIU/L, Tg-Ab: 5 kU/L, and TRAb: 0.15 IU/L). Autoantibodies were considered positive if levels were ≥ 60 kU/L for TPO-Ab and Tg-Ab and ≥ 1 IU/L for TRAb according to the locally applied cut-offs. The local reference interval for TSH in non-pregnant adults (0.3–4.5 mIU/L) was used for classification of thyroid function status postpartum. Thus, hyperthyroidism was defined as TSH < 0.3 mIU/L, hypothyroidism as TSH > 4.5 mIU/L, and a biphasic course as hyperthyroidism followed by hypothyroidism. In a subgroup of cases (n = 7), the biochemical assessment revealed an initial hypothyroid or biphasic phase followed by a hyperthyroid episode, however, individual review of cases indicated that hyperthyroidism developed after initiation of treatment with Levothyroxine, and the hyperthyroid phase was not included in the classification of the course of disease. A subclassification of overt and subclinical disease was performed according to levels of total T4 (local applied reference interval in non-pregnant adults: 60–140 mol/L). In the North Denmark Region, total thyroid hormone concentrations are used for patient care in hospital and in general practice.

### Statistical analyses

Continuous data was described with median and 95% confidence intervals (CI), and categorical variables were presented with number of cases (n) and frequency (%) and compared using the Chi-squared test. According to local data regulations, variables with less than three cases were presented as < 3. The prevalence of O905-diagnoses in Denmark from 1997 to 2016 was calculated as the number of registered cases per year divided by the number of live births recorded each year. Data were registered in Research Electronic Data Capture (REDCap®, Vanderbilt University, Tennessee, USA) and hosted at Aalborg University Hospital^11,12^. Data analyses were performed using Stata® 18.0 (StataCorp LLC, Texas, USA).

## Results

### The postpartum cohort

**Figure 2:**
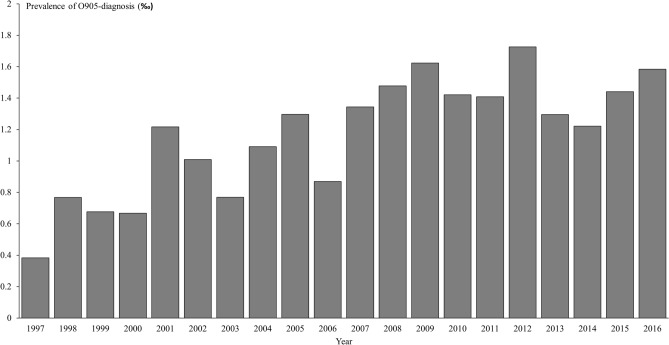
Prevalence of O905-diagnoses registered in Danish hospitals from 1997 to 2016.

Altogether 1,105 women with an O905-diagnosis of PPT were identified in the DNHR in Denmark during the 20-year period from 1997 to 2016 and, additionally, 40 women were registered in the North Denmark Region in the four-year period from 2016 to 2019 (Table 1). Nationally, the prevalence of an O905-diagnosis increased during the study period (Fig. 2) and was approximately 1.5‰ in the most recent years. The number of cases identified in the North Denmark Region (an average of 10 per year from 2016 to 2019) agreed with the overall nationwide frequency since the North Denmark Region approximates 10% of the Danish population. The regional group of women did not differ regarding maternal age and smoking; however, these women were more often overweight and nulliparous (Table 1). A small group of the women had previous registrations of thyroid disease but did not receive any thyroid medication prior to or during the pregnancy under study.

**Table 1:** Characteristics of patients diagnosed with postpartum thyroiditis (O905) in the North Denmark Region from 2016 to 2019 and in all regions of Denmark from 1997 to 2016.

	North Denmark region 2016–2019		All Danish regions 1997–2016	
	n	%	n	%
Patients	40		1,105	
Age^a^				
< 30 years	17	45.9	436	44.7
≥ 30 years	20	54.1	539	55.3
Smoking^a,b^				
Yes	5	13.5	99	10.4
No	32	86.5	856	89.6
Pre-pregnancy BMI^a^				
< 25 kg/m^2^	20	54.1	497	71.4
≥ 25 kg/m^2^	17	45.9	199	28.6
Parity^a^				
Nullipara	21	56.8	511	50.6
Multipara	16	43.2	499	49.4
Known thyroid disease	3	7.5	135	12.2
Non-thyroid autoimmune disease	< 3	NA	60	5.4

*BMI* Body mass index, *NA* Not applicable.

^a^Missing data not included: Age (n = 133), smoking (n = 153), pre-pregnancy BMI (n = 412), parity (n = 98).

^b^Current or previous smoking.

**Figure 3:**
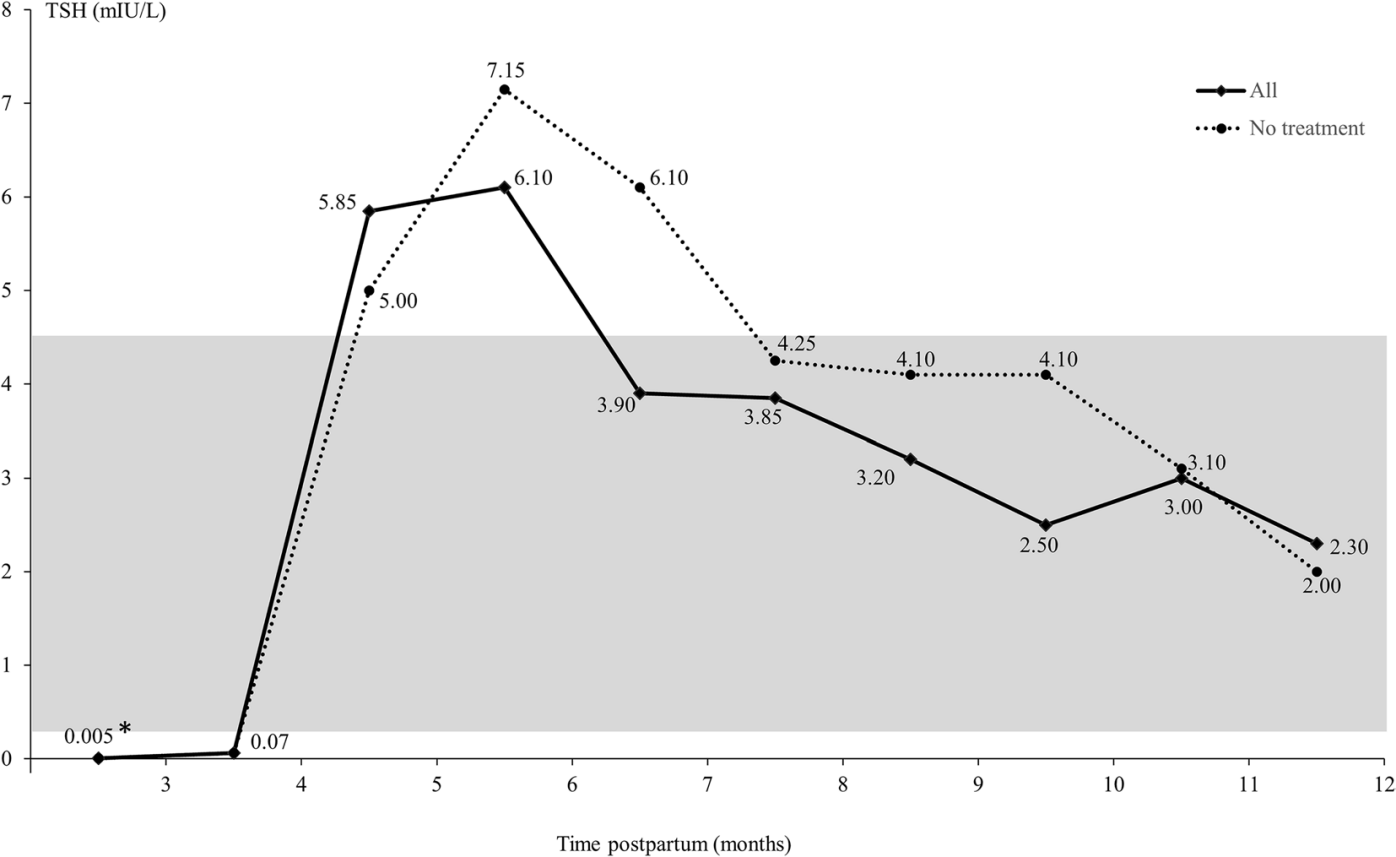
Course of thyrotropin (TSH) during the postpartum period among patients with an O905-diagnosis in the North Denmark Region. Results are illustrated by the median for each month, however, combined for the first three months (*). The solid line illustrates TSH levels among all patients and the dotted line represents women without Levothyroxine treatment at the time of blood sampling. The grey area marks the reference interval for TSH (0.3–4.5 mIU/L).

More details on the O905-diagnosis were available for the 40 women identified in the North Denmark Region from 2016 to 2019 (Table 2). In all cases, the diagnosis was registered during a visit in the Department of Endocrinology and in most cases, it was a secondary diagnosis obtained 3–12 months after birth of the child (Table 2). Biochemically, abnormal thyroid function test results were revealed in all cases, and overall initial hyperthyroidism was followed by hypothyroidism (Fig. 3 and Table 3). However, at an individual level, it differed whether the course of disease was biphasic, hyperthyroid, or hypothyroid alone (Table 2). Among patients with available measures of total T4 (n = 36), all cases had at least one biochemical assessment during follow-up at which the thyroid function abnormality was overt. Information on treatment with thyroid medication in the postpartum period showed that 27 of the 40 patients received treatment with either Levothyroxine or ATD and a total of three patients received both types of thyroid medication within the two-year follow-up period. In the second year postpartum 19 patients still received treatment with Levothyroxine. When the thyroid function test results were evaluated after the exclusion of women who received Levothyroxine at the time of blood sampling, the dynamics in thyroid function were comparable to the main analysis (Fig. 3 and Table 3).

**Table 2:** Disease characteristics of patients registered with a diagnosis of postpartum thyroiditis (O905) in the North Denmark Region from 2016 to 2019.

	n	%
Patients	40	
Type of diagnosis		
Primary diagnosis	7	17.5
Secondary diagnosis	33	82.5
Time from childbirth to diagnosis		
< 3 months	< 3	NA
3–6 months	20	50.0
6–9 months	10	25.0
9–12 months	8	20.0
12–24 months	< 3	NA
Medical treatment postpartum		
Levothyroxine	22	55.0
ATD	8	20.0
None	13	32.5
Course of the disease postpartum^a^		
Hyperthyroidism	8	20.5
Hypothyroidism	16	41.0
Biphasic^b^	15	38.5
Autoantibody positivity postpartum^c^		
TPO-Ab^a^	29	74.4
Tg-Ab^a^	21	63.6
TRAb^a^	8	20.5

*ATD* Antithyroid drugs, *TPO-Ab* Thyroid peroxidase antibodies, *Tg-Ab* Thyroglobulin antibodies, *TRAb* Thyrotropin receptor antibodies, *NA* Not applicable.

^a^Missing data not included: Course of the disease (n < 3), TPO-Ab (n < 3), Tg-Ab (n = 7), TRAb (n < 3).

^b^Hyperthyroidism followed by hypothyroidism.

^c^Assessed as minimum one value above the cut-off within the study period.

**Table 3:** Thyroid function test results in stratified time intervals after the pregnancy among patients diagnosed with postpartum thyroiditis (O905) in the North Denmark Region from 2016 to 2019. Results are illustrated for all patients with available biochemical test results (upper part) and for patients who did not receive treatment with Levothyroxine at the time of biochemical assessment (lower part).

	Ref	< 3 months			3–6 months			6–9 months			9–12 months			12–24 months		
		n	median	95% CI	n	median	95% CI	n	median	95% CI	n	median	95% CI	n	median	95% CI
All																
TSH (mIU/L)	0.3–4.5	8	0.005	0.005–1.9	27	6.0	4.4–12.4	35	3.2	2.0–4.6	28	2.3	1.6–3.1	35	1.5	1.1–2.1
Total T3 (nmol/L)	1.1–2.5	6	3.1	1.7–5.0	23	1.6	1.4–1.7	30	1.5	1.4–1.7	23	1.6	1.4–1.8	27	1.6	1.5–1.6
Total T4 (nmol/L)	60–140	6	186	98–226	25	69	57–83	31	80	76–84	22	89	83–97	26	94	95–104
No treatment																
TSH (mIU/L)	0.3–4.5	8	0.005	0.005–1.9	27	8.2	3.2–23.1	24	4.4	2.2–18.5	11	3.0	1.5–4.9	15	1.6	1.1–2.8
Total T3 (nmol/L)	1.1–2.5	6	3.1	1.7–5.0	22	1.7	1.3–1.8	16	1.6	1.4–2.1	7	1.6	1.2–2.3	9	1.6	1.2–1.8
Total T4 (nmol/L)	60–140	6	186	98–226	25	57	44–83	20	65	54–80	7	83	57–96	8	93	73–108

*Ref.* Reference interval in the local laboratory, *CI* confidence interval, *TSH* Thyrotropin, *T3* Triiodothyronine, *T4* Thyroxine.

Lastly, thyroid autoantibody status was evaluated, and around 75% of the 40 women studied were positive for TPO-Ab postpartum (Table 2). Furthermore, eight patients had levels of TRAb above the assay-specific cut-off, however, concentrations were slightly above the limit (1.0–1.7 IU/L) in seven of these patients, while a TRAb value above 4 IU/L was seen in one patient. The course of disease postpartum among TRAb-positive women was hyperthyroidism (n = 4), hypothyroidism (n = 2), or biphasic (n = 2), and three patients received ATD within the two-year follow-up period.

### The pregnancy cohort

Altogether 89 patients with a thyroid diagnosis in relation to obstetrics were studied (Table 4). Maternal characteristics were overall comparable as to whether a diagnosis of O992B (hypothyroidism) or O992C (hyperthyroidism) was registered, however, maternal smoking was more commonly seen among women with an O992C-diagnosis, and women with a diagnosis of O992B had more often received fertility treatment prior to the pregnancy (Table 4). Both diagnoses were primarily used during the woman’s visit in the Department of Gynecology and Obstetrics and were in most cases registered as a secondary diagnosis. Even if the diagnosis was used during an obstetric visit, most patients had parallel visits in the Department of Endocrinology (92% of patients for the O992B-diagnosis and 80% of patients for the O992C-diagnosis).

**Table 4:** Patient and disease characteristics among patients with an O992B- or O992C-diagnosis.

	O992B		O992C		*p*^a^
	n	%	n	%	
Patients	50		39		
Age^b^					
< 30 years	14	29.2	13	33.3	0.59
≥ 30 years	34	70.8	25	66.7	
Smoking^b,c^					
Yes	5	10.2	10	26.3	0.049
No	44	89.8	28	73.7	
Pre-pregnancy BMI^b^					
< 25 kg/m^2^	19	39.6	15	40.5	0.93
≥ 25 kg/m^2^	29	60.4	22	59.5	
Parity					
Nullipara	24	48.0	19	48.7	0.95
Multipara	26	52.0	20	51.3	
Fertility treatment	12	24.0	3	7.7	0.04
Family history of thyroid disease	13	34.2	17	53.1	0.11
Type of diagnosis					
Primary diagnosis	4	8.0	5	12.8	0.45
Secondary diagnosis	46	92.0	34	87.2	
Place of diagnosis^a^					
Department of Gynecology and Obstetrics	46	92.0	35	89.7	0.71
Department of Endocrinology	4	8.0	4	10.3	
Diagnosis of thyroid disease					
Hyperthyroidism	< 3	NA	25	64.1	< 0.01
Hypothyroidism	45	90.0	6	15.4	< 0.01
None	< 3	NA	6	15.4	0.62
Onset of thyroid disease prior to pregnancy	47	94.0	23	59.0	< 0.01
Medical treatment prior to pregnancy	45	90.0	19	48.7	< 0.01
Onset of thyroid disease during pregnancy	< 3	NA	10	25.6	< 0.01
Medical treatment during pregnancy^b^	45	91.8	15	38.5	< 0.01
Levothyroxine	45	91.8	8	20.5	< 0.01
ATD	< 3	NA	8	20.5	< 0.01

*BMI* Body mass index, *ATD* Antithyroid drugs, *NA* Not applicable.

^a^p-value is the result of comparison using Chi-square test.

^b^Missing data not included: Age (n = 3), smoking (n < 3), pre-pregnancy BMI (n = 4), family history of thyroid disease (n = 19), medical treatment during pregnancy (n < 3).

^c^Current or previous smoking.

From the review of medical records, it was identified that 81 of the 89 patients studied (91.1%) suffered from thyroid disease. Stratified by type of diagnosis (Table 4), the frequency of suffering from hypothyroidism among patients with a diagnosis of O992B was higher compared to the frequency of suffering from hyperthyroidism among patients with a diagnosis of O992C. A total of 12 patients (30.8%) with an O992C-diagnosis were misdiagnosed as they either suffered from hypothyroidism or had no thyroid disease. A notable difference between patients diagnosed with O992B and O992C, respectively, was the onset of disease in relation to the pregnancy under study. Nearly all patients with O992B had onset of disease prior to the pregnancy, whereas for O992C a fourth of patients were first-time diagnosed during the pregnancy (Table 4). Regarding medical treatment, the findings similarly differed according to the specific diagnosis. Thus, more than 90% of patients with an O992B-diagnosis received treatment with Levothyroxine, whereas less than half of the patients with an O992C-diagnosis received treatment during the pregnancy with either Levothyroxine or ATD (Table 4).

## Discussion

### Principle findings

This is the first study on the validity of obstetric and postpartum thyroid ICD-10 diagnoses. PPT was verified in 39 of 40 cases with a positive predictive value (PPV) of 97.5% for a diagnosis of O905. Regarding the obstetric diagnoses, the validity was lower, and 90.0% of patients with an O992B-diagnosis correctly had hypothyroidism, whereas hyperthyroidism was verified in 64.1% of patients with an O992C-diagnosis.

### Interpretation

Our study used information from the Danish nationwide health registers with a general high completeness and validity, however, reported PPV for different diagnoses range from less than 15% and up to 100%^7^. Regarding the diagnoses of hyperthyroidism and hypothyroidism, a study in non-pregnant adults reported misclassification in less than 2% of cases registered in the DNHR^13^, while the diagnosis of subacute thyroiditis was found to be correct in only 62% of cases^14^. In comparison, our study in female patients and with a focus on thyroid disease diagnosed in relation to pregnancy and the postpartum period identified a PPV in the range from 64.1% to 97.5%. The PPV was lower for the obstetric diagnoses than for PPT being 90.0% and 64.1% for obstetric hypothyroidism and hyperthyroidism, respectively. Coding errors e.g., in the form of mix-up between an O992B- and O992C-diagnosis may have contributed and registration of O992-diagnoses in patients without thyroid disorders was seen. As for smoking, around 10% of patients with obstetric hypothyroidism were either former or current smokers during pregnancy while it was the case for a fourth of patients registered with obstetric hyperthyroidism. The latter corresponds to existing literature as smoking increases the risk of developing GD^15^. In comparison, the frequency of maternal smoking during pregnancy in general declined in recent years, and 8.2% of Danish women smoked or quitted smoking during pregnancy when evaluated in 2019^16^. Our sample size was limited and did not allow for further stratification. Thus, we could not draw definite conclusions regarding smoking as a protective factor of hypothyroidism which has previously been proposed^15,17^.

Regarding fertility treatment, a significant difference was found between women diagnosed with obstetric hypothyroidism and hyperthyroidism, respectively. Thus, approximately one in four of patients with obstetric hypothyroidism had undergone fertility treatment while it was the case for around 8% of patients registered with obstetric hyperthyroidism. In comparison 9.2% of children born in 2019 were conceived by assisted reproduction^18^. In general, symptoms of hypothyroidism are often unspecific, and the disorder may be undetected^19^. Screening for thyroid disease is recommended in women undergoing fertility treatment^6^. Thus, we speculate if hypothyroidism is more often detected in these cases considering the unspecific symptoms. Following this line of thought, the higher rate of fertility treatment among patients diagnosed with obstetric hypothyroidism may be related to confounding by indication.

In our study, the observed prevalence of PPT was low compared to existing literature. We found a prevalence of approximately 1.5‰ in the most recent years which may be explained by the fact that the DNHR does not capture the total number of PPT cases, but only cases managed in hospital. In comparison, the global prevalence of PPT is around 5%, however, with a large variation across studies and reported prevalence ranging from 1.1 to 16.7%^6^. Our study showed an increase in the prevalence of the O905-diagnosis from 1997 and onwards, but up until 1994, when ICD-10 was introduced in Denmark, a specific diagnosis of PPT did not exist. This may contribute to the initial increase in number of patients registered during our study period as part of the adaption to the ICD-10-coding rather than an actual increase in diseased cases.

In the regional postpartum cohort, half the patients were registered with an O905-diagnosis 3–6 months after birth of the child. The biphasic course or isolated hyperthyroidism typically have an onset 2–4 months postpartum while isolated hypothyroidism presents 3–7 months postpartum^4^. Thus, in our study cohort the patients were more often registered with the diagnosis later than the general figures. Our study was register-based, hence, onset of disease was determined from the date the diagnosis was registered which is an indirect measure of disease onset. Thereby, abnormal thyroid function or symptoms may have been present at an earlier stage. Furthermore, a diagnostic delay may occur in some cases, for example if the symptoms of PPT are interpreted as part of the physical and emotional stress of being a mother with a newborn child^3^.

Reports on the course of the disease vary with half of PPT patients presenting with hypothyroidism alone, a fourth with isolated thyrotoxicosis, and a fourth with the classic biphasic presentation^1^. This is not completely consistent with our findings as ~ 40% of patients had isolated hypothyroidism and ~ 40% had a biphasic course while around 20% presented with thyrotoxicosis alone. However, other studies have shown results in line with our observations^20^.

Development of PPT is associated with the presence of thyroid antibodies, especially TPO-Ab and Tg-Ab, and women who are TPO-Ab positive in the first trimester have a risk of 33–50% for developing PPT^6^. In our study, approximately 75% of patients were positive for TPO-Ab postpartum which agrees with the literature^2^. We found that eight patients were positive for TRAb when evaluated from a cut-off of 1.0 IU/L, although PPT patients typically are negative for TRAb. However, it has been reported in other studies that up to 25% of women with PPT had detectable TRAb^21^. In our cohort, seven of the eight TRAb positive patients presented with low titers of TRAb in the range from 1.0 to 1.7 IU/L and according to the manufacturer of the assay, results in the range from 1.0 to 1.5 IU/L are considered as a grey zone. This led us to conclude that only the patient with consistently and highly elevated TRAb postpartum was compatible with a diagnosis of GD.

ATD should not be used for treatment of PPT^6^. In our study cohort, it was observed that eight of the patients with a diagnosis of PPT were treated with ATD in the postpartum period and only three of these patients had positive TRAb status and in low titers (1.0–1.7 IU/L). This is a concern; however, the diagnosis may be difficult at initial patient presentation which may cause a misdiagnosis of hyperthyroidism. This has also been found for other subtypes of thyroiditis, e.g. subacute thyroiditis^14^. In our study, 22 patients (55%) with PPT received Levothyroxine, and 19 patients still redeemed prescriptions of the drug in the period from 12 to 24 months postpartum. Furthermore, one third of the patients with TSH assessment within the second year postpartum had results outside the reference interval suggesting that thyroid function abnormalities were still present. In general, it is described that most women with PPT return to an euthyroid state within the first postpartum year^1^. However, an Italian study has proposed that a re-evaluation of this fact is warranted due to observations of permanent hypothyroidism in up to 54% of cases 12 months postpartum^22^.

### Methodological comments

Our study population was identified from the DNHR, which may have increased the risk of referral bias, as the register only covers patients diagnosed in hospital^23,24^. In general, the risk of referral bias increases with age among Danish patients, thus, mainly the younger patients with thyroid disease are referred to hospital for management of the disease^23,24^. Furthermore, it is recommended that women with thyroid disease who become pregnant are referred to a hospital endocrine specialist unit as soon as the pregnancy is detected. Thus, we find it unlikely that referral bias influenced our findings in the pregnancy cohort. On the other hand, PPT may be managed in general practice alone, and our postpartum cohort may have a higher severity of the disease or more comorbidities which limits the generalizability of our findings regarding this diagnosis. A strength of the study was that all patients in the North Denmark Region registered with O905 from 2016 to 2019 as well as O992C in 2019 were included without any selection. However, even if all patients were included the numbers were small in some stratified analyses. Data were gathered from registries as well as medical records and were collected without contact to the patients or physicians, however, possible information bias is considered non-differential. For diagnosis registration, it is important to be aware of potential differences in coding practices, e.g., differences in the interpretation of the O992-diagnoses due to the wording of the diagnosis code. This may leave some uncertainty as to whether all pregnant women with a verified thyroid disease should be registered with the diagnosis, or it should be used solely in cases with complications due to thyroid disease. Finally, the use of total thyroid hormone concentrations in the North Denmark Region contrasts with the more general use of free thyroid hormone concentrations, especially in pregnant women^25^. However, the total thyroid hormone measures are provided together with a measure of thyroid binding globulin, and the clinicians are familiar with these measures. Thus, this practice is not expected to influence the reported validity of the diagnosis studied.

## Conclusion

This is the first study to validate ICD-10 diagnoses of obstetric and postpartum thyroid diseases in the DNHR. Overall, a high validity was seen for the diagnosis of PPT (O905) and obstetric hypothyroidism (O992B) with a PPV of 97.5% and 88.0%, respectively, whereas for obstetric hyperthyroidism (O992C) the diagnosis could not be verified in one third of the cases. The findings have clinical and scientific implications, and further studies in different populations are warranted to corroborate and extend the findings.

## Data Availability

The data cannot be shared due to regulatory restrictions that apply to the availability of data generated and analyzed during this study to preserve patient confidentiality and according to the GDPR regulations.
